# Study on Capillary Water Absorption of Waterborne-Polyurethane-Modified Recycled Coarse Aggregate Concrete

**DOI:** 10.3390/polym15193860

**Published:** 2023-09-22

**Authors:** Guoxi Fan, Wantong Xiang, Jing Yang, Shutong Yang, Chunping Xiang

**Affiliations:** 1 College of Engineering, Ocean University of China, Qingdao 266100, China; fanguoxi@ouc.edu.cn (G.F.); wtxiang101@163.com (W.X.); yangjing2416@163.com (J.Y.); 2 State Key Laboratory for Strength and Vibration of Mechanical Structures, School of Aerospace Engineering, Xi’an Jiaotong University, Xi’an 710049, China

**Keywords:** waterborne polyurethane, curing system, polymer-cement ratio, capillary water absorption, pore structure

## Abstract

The reuse of construction and demolition waste as a substitute for natural coarse aggregate in the production of recycled concrete has been widely used. In order to study the capillary water absorption performance of waterborne-polyurethane-modified recycled aggregate concrete (WPUMRC), the effects of different curing systems, polymer-cement ratios, and waterborne polyurethane addition methods on the cumulative water absorption and the rate of capillary water absorption of WPUMRC were analyzed, and through MIP tests, the micro modification mechanism of waterborne polyurethane in recycled concrete was analyzed. The results indicate that the optimal curing system for both DC (waterborne polyurethane is added separately from water) and HC (waterborne polyurethane is mixed with some effective water and then added) is the 14 d standard curing—14 d indoor natural drying curing system. Waterborne polyurethane can fill the pores and micro-cracks inside WPUMRC or interweave with the hydration products of cement to form a spatial network structure, reducing the porosity, and thereby improving the capillary water absorption performance of WPUMRC. Based on the MIP test results, the grey correlation method was used to establish the relationship between capillary water absorption and the pore structure of WPUMRC under the optimal curing system. In addition, the prediction model of capillary water absorption in recycled concrete was established according to the test results, which can be used to predict WPUMRC’s capillary water absorption performance.

## 1. Introduction

Concrete material is the most widely used building material in the world. Large amounts of coarse and fine aggregates are required to prepare concrete, while the consumption of coarse and fine aggregates has applied enormous pressure on the natural environment. Meanwhile, a large amount of construction and demolition (C&D) waste is generated with the rapid development of the construction industry. According to statistical data in 2015, 2.5 billion tons of C&D waste had been generated in China [1]. In the past, most C&D waste was dumped or landfilled due to improper management. This treatment causes the occupation of a large amount of land resources, which seriously affects the ecological environment [2]. As a result, it is crucial to deal with C&D waste reasonably and then reduce the pressure on the natural environment. Fortunately, 80% of C&D waste is composed of concrete, blocks, and bricks, which are prone to being recycled [3]. Research also shows that most C&D waste can be reused as building materials [4], which provides an effective way to save resources and reduce pressure on the natural environment.

Generally, C&D waste can be processed into recycled coarse aggregate through recycling, sorting, crushing, grading, and other treatment methods, and then prepared into recycled coarse aggregate concrete (RAC). It provides an effective means for the reuse of C&D waste. However, there are many defects in recycled coarse aggregate concrete, such as more microcracks, a looser interface, higher porosity, and so on. Compared with ordinary concrete, the durability, the frost resistance, and some mechanical properties of recycled concrete are poorer [5]. Consequently, the working performance of recycled concrete is required to be improved. Currently, many technologies for improving the working performance of recycled concrete have been proposed, such as microwave-assisted recycling technology [6,7,8], particle shaping technology [9], pre-foaming with acid [10,11], silane-based surface treatment [12], the two-stage mixing approach [13,14], the mixed water compensation method [15], and adding composite materials [16,17,18,19]. Among these, one of the effective methods is to add polymers to the concrete.

In view of the obvious modification effect of polymers on concrete, extensive research on polymer concrete has been carried out. Previous studies show that polymer-modified concrete has stronger adhesion, better flexibility, and chemical resistance [20]. When referring to polymer-modified concrete, frequently used polymer modifiers include latex, powder, and resin. The working performance of polymer-modified concrete is affected by the kind and content of the polymer [21,22]. The modification effects of three kinds of polymers, i.e., styrene-butadiene rubber latex, polyacrylic ester emulsion, and an organic silicon waterproof agent, on ordinary concrete were analyzed. The results showed that the addition of the polymers improved the impermeability of the concrete, while the improvement degree varied with the different kinds and contents of the polymers [23]. SBR and epoxy latex were mixed into ordinary concrete, and a new concrete-polymer composite material with excellent compressive properties, tensile properties, frost resistance, wear resistance, and impermeability was obtained with the optimal content of polymer [24]. With the addition of SBR latex, the strength, bending toughness, and impact toughness of concrete have been greatly improved [25]. Among many polymer modifiers, polyurethane has attracted more attention due to its better designability [26]. In recent years, considering environmental protection and green development, waterborne polyurethane has gradually replaced solvent-based polyurethane. As a new polymer material, waterborne polyurethane has better adaptability to cement. A waterborne polyurethane with relatively lower cost and higher solubility in water was adopted to improve the performance of ordinary concrete. The findings demonstrated that the appropriate content of the waterborne polyurethane increased the elastic modulus, compressive strength, splitting tensile strength, and flexural strength of the waterborne-polyurethane-modified concrete. Furthermore, it could effectively improve the chloride penetration resistance and impermeability of the concrete after the waterborne polyurethane was added [27]. However, there are few studies on waterborne-polyurethane-modified recycled concrete with different contents of waterborne polyurethane.

Meanwhile, the mechanical properties and durability of concrete depend on its curing system. Previous studies have shown that the compressive strength of concrete increases with increases in the wet curing time [28]. The flexural and tensile strength of concrete under immersion curing conditions is higher than that under natural curing conditions [29]. The curing condition of a higher relative humidity of up to 90% leads to better mechanical properties in the interfacial transition zone of concrete [30]. A similar conclusion has been found in terms of durability. The initial water absorption, permeability, and carbonization depth of RAC cured in water are lower than those of RAC cured in air [31]. It facilitates the adequate hydration of cement under appropriate conditions of moisture and temperature, while it is not conducive to the strength of concrete under excessive humidity [32]. The hydration of cement can be promoted in a humid environment, while the formation of a bonding film between the polymer and the cementitious material can be promoted in a dry environment [33]. With that in mind, some scholars investigated the performance of concrete using a mixed curing system. The research shows that the strength of RAC using a mixed wet and dry curing system can be effectively improved [34]. A mixed curing system can also promote the bonding between the plastic waste aggregate and binder [35]. Similarly, there are few studies on waterborne-polyurethane-modified recycled concrete under different curing systems.

In view of this, the capillary water absorption of waterborne-polyurethane-modified recycled concrete (WPUMRC) was taken as the research object. A waterborne polyurethane with relatively higher solubility in water was adopted to improve the performance of recycled coarse concrete. Then, the capillary water absorption performance of WPUMRC under different curing systems, polymer-cement ratios, and addition methods was investigated. In addition, the micro modification mechanism of WPUMRC was also analyzed. Finally, theoretical predictions were made regarding the capillary water absorption performance of WPUMRC.

## 2. Experimental Program

### 2.1. Material Characteristics

Ordinary Portland P·O 42.5 cement was used as the cementitious material, which is detailed in Table 1. Natural river sand with a size of less than 5 mm was used as the fine aggregate, which was naturally air-dried. Obtained from C&D waste, recycled coarse aggregate (RA) with a size of 5~20 mm was used as the coarse aggregate, which was also naturally air-dried. The recycled coarse aggregate was screened according to GB/T 25177-2010 [36]. The physical properties of the recycled coarse aggregate are shown in Table 2. The basic parameters of the waterborne polyurethane emulsion are shown in Table 3. The polymer emulsion contained a surfactant, which makes it easy to introduce a large number of bubbles in the process of mixing and vibrating into the concrete. It is necessary to add an appropriate amount of a defoamer to eliminate air bubbles formed during the mixing process. An antifoaming agent is an admixture that can reduce the surface tension of a liquid, thereby reducing or eliminating existing air bubbles [37]. An organosilicon antifoaming agent was used, the basic information of which is shown in Table 4. In order to reduce water consumption and improve the fluidity of the WPUMRC, a polycarboxylate superplasticizer was also added.

### 2.2. Mix Design

The mix design of the WPUMRC (waterborne-polyurethane-modified recycled aggregate concrete) with different polymer-cement ratios is shown in Table 5. Additional water was needed for the WPUMRC due to the higher water absorption in the recycled coarse aggregate. The moisture content in the recycled coarse aggregate should not reach a fully saturated state. Otherwise, the effective interface transition zone between the recycled coarse aggregate and the new cement paste will fail [38]. Therefore, the additional water consumption should be calculated based on the recycled coarse aggregate in a saturated surface dry state. A recycled coarse aggregate in a saturated surface dry state neither absorbs water from the recycled concrete nor releases water into the recycled concrete mixtures. The additional water consumption was determined via mix proportion adaptation. The dosages of the waterborne polyurethane emulsion were 0 (i.e., the control group), 0.5%, 1.0%, 1.5%, and 2.0% of the mass of cement. The mass of the organosilicon antifoaming agent was 4% of the mass of the waterborne polyurethane emulsion. The water in the waterborne polyurethane and antifoaming agent should be taken out of the total effective water of the WPUMRC. Before use, the waterborne polyurethane emulsion should be fully shaken to prevent flocculation.

### 2.3. Preparation and Curing of Specimens

In view of the fact that the two-stage mixing approach (TSMA) can improve the strength and durability of RAC by increasing its bonding in the interfacial transition zone (ITZ) of the recycled aggregate [38,39], the WPUMRC was prepared using the two-stage mixing approach. In the first stage, the recycled coarse aggregate was uniformly mixed into the concrete mixer. Then, the additional water was slowly poured into the concrete mixer and fully mixed until the recycled coarse aggregate reached a saturated surface dry state. In the second stage, the river sand and cement were poured into the concrete mixer and uniformly mixed. Then, the effective water, waterborne polyurethane, and other admixtures were slowly poured into the concrete mixer. In this process, two different waterborne polyurethane addition methods were adopted. In one method, the effective water and the waterborne polyurethane were separately added into the concrete mixer (DC). In the other method, the partially effective water and the waterborne polyurethane were first mixed together and then added to the concrete mixer (HC). For DC, the waterborne polyurethane locally adhered to the surface of the WPUMRC mixture in the form of aggregates. For HC, waterborne polyurethane particles were evenly dispersed with water inside the WPUMRC mixture. After that, the fresh concrete mixture was poured into the corresponding molds.

The hydration of cement requires an appropriate amount of water, while the curing of waterborne polyurethane requires a dry environment [40]. To investigate the impact of the curing system on the capillary water absorption of WPUMRC, five curing systems were adopted, i.e., 0 d standard curing—28 d indoor natural drying curing (0–28), 3 d standard curing—25 d indoor natural drying curing (3–25), 7 d standard curing—21 d indoor natural drying curing (7–21), 14 d standard curing—14 d indoor natural drying curing (14–14), and 28 d standard curing—0 d indoor natural drying curing (28–0). Among these, the condition of standard curing corresponded to a temperature of 20 ± 2 °C and a humidity of greater than 95%. The condition of indoor natural drying curing corresponded to a temperature of 20 ± 4 °C and a humidity of 40~65%.

### 2.4. Test Methods

#### 2.4.1. Capillary Water Absorption Test

**Figure 1:**
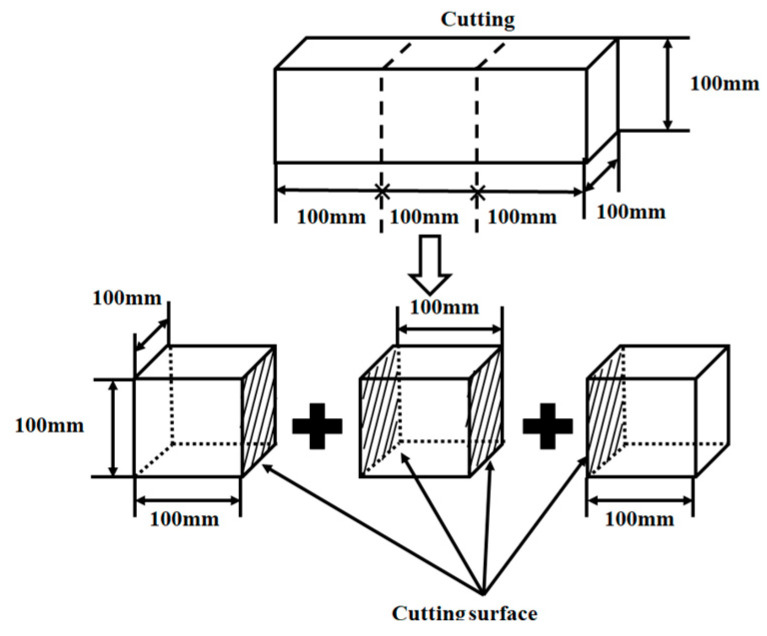
Cutting process.

**Figure 2:**
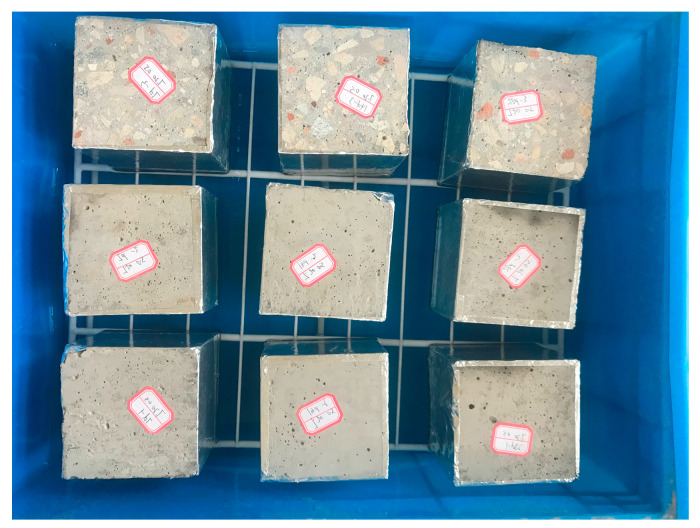
The device of capillary water absorption.

Prism specimens with dimensions of 100 mm × 100 mm × 300 mm were prepared, and then cut into three groups of cube specimens. The cutting process is shown in Figure 1. Before conducting the capillary water absorption test, the cube specimens were put into an electric blast drying oven for drying. The drying temperature was set at 70 °C in order to avoid aging and softening the waterborne polyurethane under a continuous high temperature. After continuous drying for 2 d, the cube specimens were taken out and naturally cooled at room temperature. Then, the four sides perpendicular to the cutting surface were sealed with aluminum foil tape to ensure the one-dimensional transmission of water [41,42]. After sealing, a standing time of 1~2 days was required to ensure a stronger adhesion between the aluminum foil tape and the cube specimens. According to ASTM C 1585 [43], a one-dimensional capillary water absorption test was conducted on the cutting surfaces of the cube specimens. The capillary water absorption test device is shown in Figure 2. The test time and tolerance time are shown in Table 6.

#### 2.4.2. MIP Test

The mercury intrusion porosimetry (MIP) test was carried out with an Auto-pore V 9600 to study the pore structure of the WPUMRC. The surface tension of mercury and contact angle were set as 0.485 N/m and 130°, respectively. The applied pressure was set from 0.1 psia to 61,000 psia in the automatic increment mode.

## 3. Analysis and Discussion of Test Results

**Figure 3:**
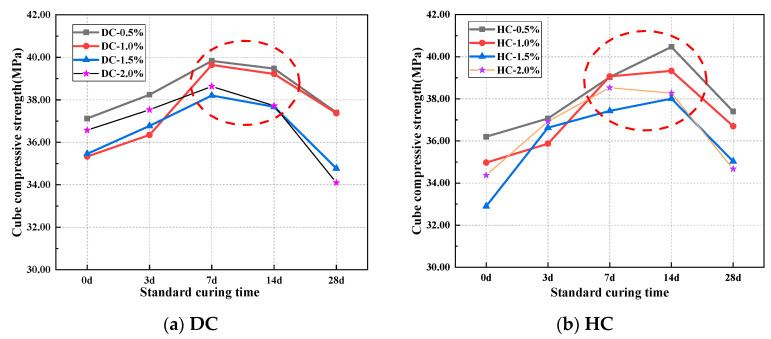
The effect of curing system on compressive strength.

The effect of the curing system on the cube’s compressive strength is shown in Figure 3. With the same polymer-cement ratio, whether DC or HC was adopted, the compressive strength of WPUMRC first increased and then decreased with the increase in the standard curing time. The curing systems of 7 d standard curing—21 d indoor natural drying curing and 14 d standard curing—14 d indoor natural drying curing are beneficial for the compressive strength of WPUMRC.

For DC, with different polymer-cement ratios, the compressive strength of the specimen under 7 d standard curing—21 d indoor natural drying curing (7–21) was increased by 5.63~12.20%, 3.89~9.05%, and 6.07~13.28% compared with that under 0 d standard curing—28 d indoor natural drying curing (0–28), 3 d standard curing—25 d indoor natural drying curing (3–25), and 28 d standard curing—0 d indoor natural drying curing (28–0), respectively. The compressive strength of the specimen under 14 d standard curing—14 d indoor natural drying curing (14–14) was increased by 3.14~11.01%, 0.51~7.90%, and 4.95~10.62% compared with that under the systems of 0–28, 3–25, and 28–0, respectively. For HC, with different polymer-cement ratios, the compressive strength of the specimen under the system of 7–21 was increased by 7.82~13.77%, 2.18~8.92%, and 4.36~11.13% compared with that under the systems of 0–28, 3–25, and 28–0, respectively. The compressive strength of the specimen under 14–14 was increased by 11.35~15.53%, 3.63~9.65%, and 7.17~10.38% compared with that under the systems of 0–28, 3–25, and 28–0, respectively.

Therefore, taking the compressive strength as the control criterion, the appropriate curing system for WPUMRC is 7 d standard curing—21 d indoor natural drying curing or 14 d standard curing—14 d indoor natural drying curing.

### 3.1. Cumulative Capillary Water Absorption 

#### 3.1.1. Effect of Curing System on Cumulative Capillary Water Absorption

**Figure 4:**
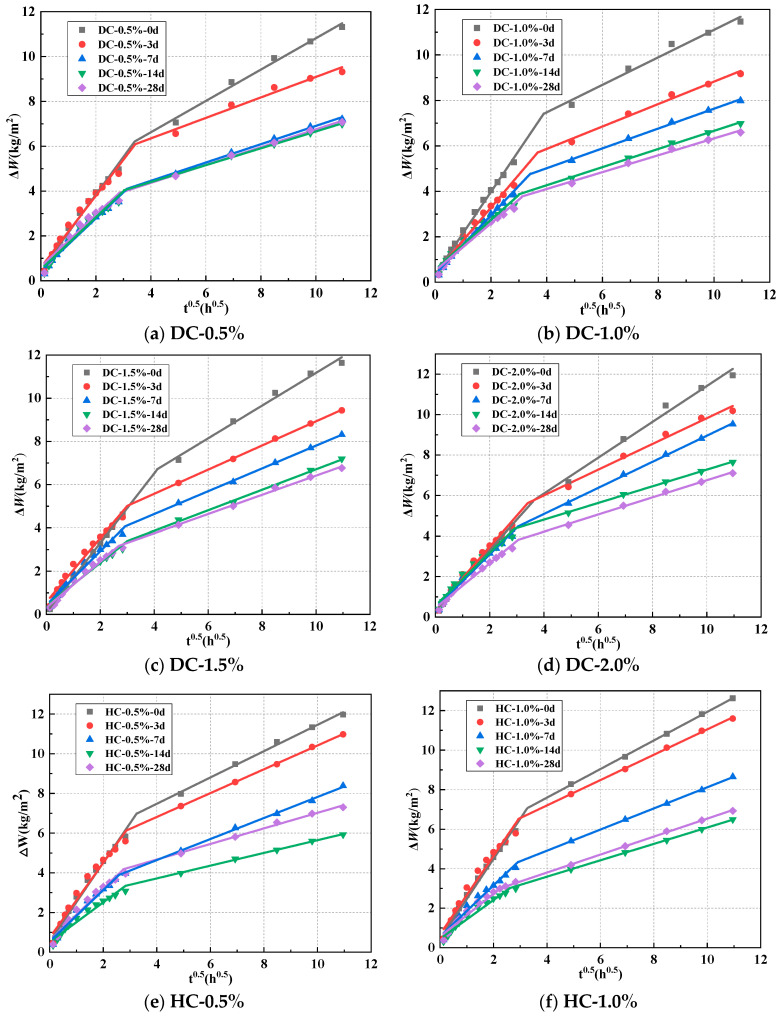

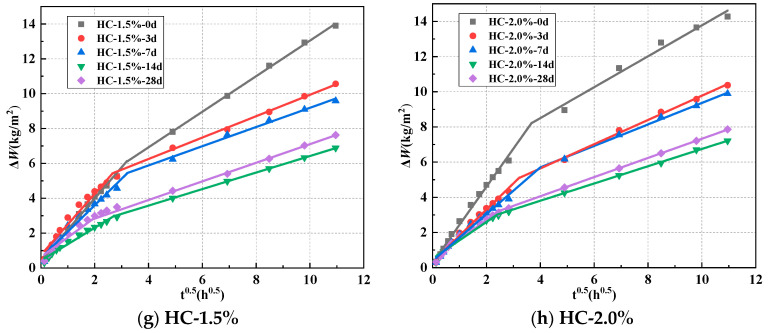
The relationship between the cumulative water absorption and curing system using different addition methods and polymer-cement ratios (**a**–**h**).

The curves of cumulative water absorption (Δ*W*) for WPUMRC under different curing systems are shown in Figure 4. Whether DC or HC was adopted, the cumulative water absorption of WPUMRC always rapidly increased in the initial stage and then slowly in the later stage. This can be attributed to the fact that in the initial stage of the capillary water absorption test, the WPUMRC is not fully saturated, and water rapidly invades under capillary forces. As the capillary water absorption test progresses, the amount of free water present within the WPUMRC increases. Consequently, the speed of water absorption in the WPUMRC slows down due to the influence of gravity.

For DC, the cumulative water absorption of the WPUMRC gradually decreased with the increase in the standard curing time. It can be seen that with the same polymer-cement ratio, the system of 28 d standard curing—0 d indoor natural drying curing had the lowest cumulative water absorption. Under the system of 14 d standard curing—14 d indoor natural drying curing, the cumulative water absorption of the WPUMRC was close to that under the system of 28 d standard curing—0 d indoor natural drying curing. This is because sufficient water and a suitable temperature were supplied to the WPUMRC under standard curing, promoting the cement’s hydration. The deeper the degree of cement hydration, the fewer pores inside the WPUMRC, and the weaker the water transfer ability. In addition, waterborne polyurethane agglomerates can solidify into films under indoor drying curing. Both waterborne polyurethane agglomerates and films can fill the pores and microcracks, reducing the pore connectivity and weakening water transport inside the WPUMRC. When the WPUMRC was placed in a dry environment after 14 d standard curing to release the water stored in the recycled coarse aggregate, it promoted continuous cement hydration. Therefore, the degree of cement hydration inside the WPUMRC was similar under the above two curing systems. In contrast, under the three curing systems of 0 d standard curing—28 d indoor natural drying curing, 3 d standard curing—25 d indoor natural drying curing, and 7 d standard curing—21 d indoor natural drying curing, the standard curing time was insufficient, and the water released from the recycled coarse aggregate was limited. As a result, the concrete lacked a water supply, the cement hydration reaction was restricted, and the capillary water absorption performance was not significantly improved.

For HC, the cumulative water absorption of the WPUMRC first decreased with the increase in the standard curing time and then increased, reaching the minimum value under the 14 d standard curing—14 d indoor natural drying curing. The reason for the above phenomenon is that sufficient water and a suitable temperature were supplied to the WPUMRC in the early standard curing, and the water stored in the recycled coarse aggregate was released in the later drying curing, which promoted continuous cement hydration, thereby increasing the complexity of the pore structure and reducing the porosity within the WPUMRC. In addition, the waterborne polyurethane particles are more evenly dispersed in the WPUMRC mixture for HC. The waterborne polyurethane particles continuously gather together to form agglomerates and solidify into films under drying curing, and the polymer films interweave with the hydration products of cement to form a spatial network structure [20]; moreover, the waterborne polyurethane agglomerates and films can fill both the pores and microcracks inside the WPUMRC, reducing the pore connectivity, and increase the tortuosity of water transport paths, thereby weakening the capillary water absorption. However, the WPUMRC is always in a humid environment under the system of 28 d standard curing—0 d indoor natural drying curing. The waterborne polyurethane particles cannot gather and solidify into films, making them unable to function.

Consequently, whether DC or HC is adopted, combined with the previous discussion about compressive strength, it can be determined that the curing system of 14 d standard curing—14 d indoor natural drying curing is optimum for WPUMRC.

#### 3.1.2. Effect of Polymer-Cement Ratio on Cumulative Capillary Water Absorption

**Figure 5:**
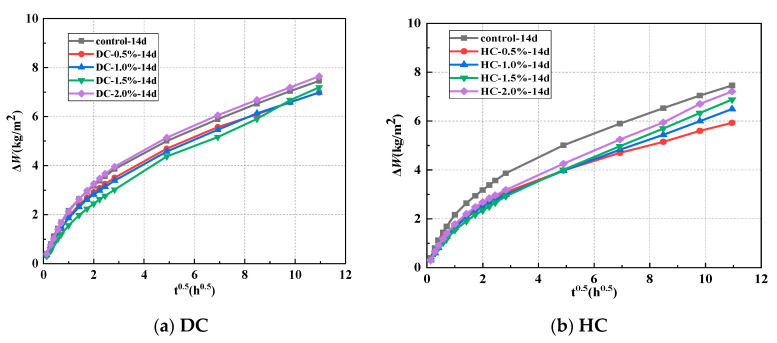
The relationship between the cumulative water absorption and polymer-cement ratio using different addition methods, (**a**) DC, (**b**) HC.

The relationship between the cumulative water absorption (Δ*W*) and the polymer-cement ratio of WPUMRC under the optimal curing system of 14 d standard curing—14 d indoor natural drying is shown in Figure 5. The capillary water absorption performance of WPUMRC was superior to that of ordinary recycled concrete except for the WPUMRC with a polymer-cement ratio of 2.0% for DC. For example, when the polymer-cement ratio was 2.0%, the cumulative water absorption values of WPUMRC for 4 h, 8 h, and 5 d were 3.25941 kg/m^2^, 3.96002 kg/m^2^, and 7.64687 kg/m^2^, respectively. Meanwhile, the corresponding cumulative water absorption values of ordinary recycled concrete were 3.17750 kg/m^2^, 3.86229 kg/m^2^, and 7.45734 kg/m^2^. For HC, waterborne polyurethane had a more significant improvement effect on the capillary water absorption performance of WPUMRC. However, with the same waterborne polyurethane addition method, the improvement effect of waterborne polyurethane at different dosages on the capillary water absorption performance of recycled concrete was not significantly different. The reasons for the above phenomenon can be attributed to the capillary water absorption performance of WPUMRC being comprehensively affected by the degree of cement hydration, the dispersion of the waterborne polyurethane, and the degree of film formation in the waterborne polyurethane. Waterborne polyurethane has a dual effect on recycled concrete.

After waterborne polyurethane is added, the partially unhydrated cement particles are wrapped or encapsulated by the waterborne polyurethane, weakening the cement hydration reaction inside the WPUMRC, thereby increasing the porosity, or coarsening the pore structure. The above phenomenon is not conducive to improving the capillary water absorption performance of WPUMRC. Meanwhile, the pores and microcracks inside WPUMRC can be filled with waterborne polyurethane agglomerates or films [42,44], reducing the pore connectivity and the pore tortuosity, or increasing the complexity of the pore structure, thereby weakening the water transport inside the WPUMRC. The above phenomenon is conducive to improving the capillary water absorption performance of WPUMRC. For DC, when the polymer-cement ratio was 2.0%, the cumulative water absorption of WPUMRC was similar to that of the control group. This is because the favorable and unfavorable effects mentioned above reached approximately a balance. In the cases of the other groups, the above favorable effects played a dominant role. As a result, the capillary water absorption of WPUMRC was improved.

#### 3.1.3. Effect of Addition Method on Cumulative Capillary Water Absorption

**Figure 6:**
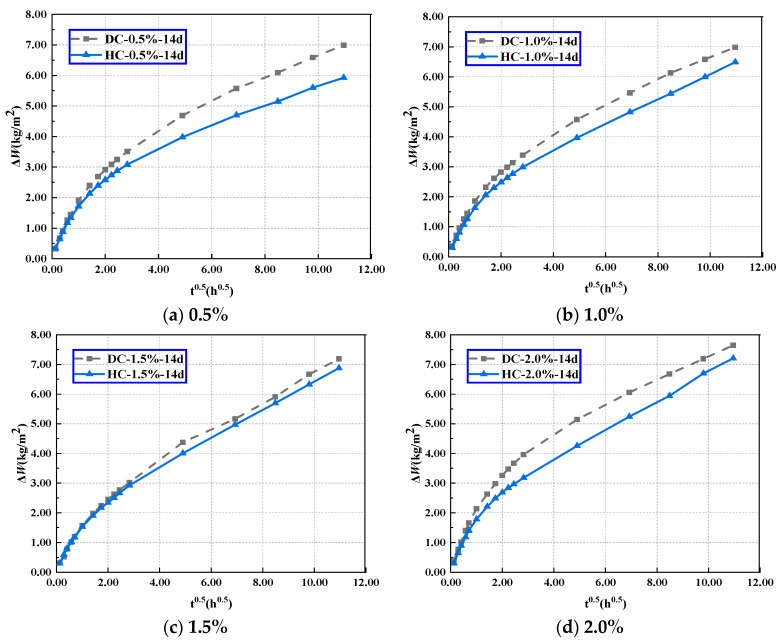
The effect of waterborne polyurethane addition method on the cumulative water absorption with different polymer-cement ratios: (**a**) 0.5%, (**b**) 1.0%, (**c**) 1.5%, (**d**) 2.0%.

The effect of the waterborne polyurethane addition method on the cumulative water absorption of WPUMRC under the optimal curing system is shown in Figure 6. It can be seen that the addition method of HC had a better improvement effect on the capillary water absorption performance of WPUMRC with the same polymer-cement ratio. This is because, at the beginning of mixing waterborne polyurethane with water and then adding it to recycled concrete, the waterborne polyurethane is dispersed inside the WPUMRC mixture with the water, resulting in a more uniform distribution and wider distribution range. This facilitates the polymer film to fill the pores and refine the pore structure in the WPUMRC, which, in turn, reduces its cumulative water absorption. Moreover, compared with DC, the encapsulation effect of waterborne polyurethane on unhydrated cement particles in HC is weaker, which is conducive to the cement hydration reaction.

### 3.2. The Rate of Capillary Water Absorption 

#### 3.2.1. Effect of Curing System on the Rate of Capillary Water Absorption

**Figure 7:**
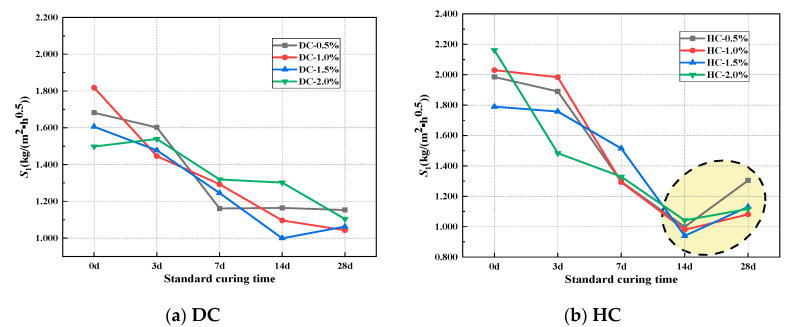
The relationship between the initial rate of absorption and curing system: (**a**) DC, (**b**) HC.

**Figure 8:**
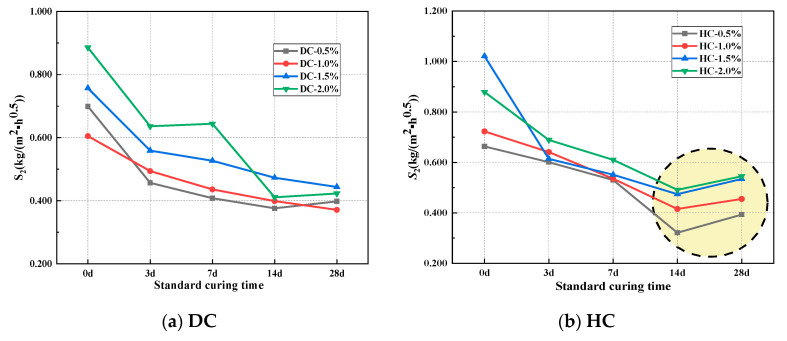
The relationship between the second rate of absorption and curing system: (**a**) DC, (**b**) HC.

The rate of capillary water absorption is an important indicator for evaluating the durability of concrete materials. The initial rate of absorption *S*_1_ is defined as the linear fitting value of the data corresponding to the first stage of the cumulative water absorption curve. The second rate of absorption *S*_2_ is defined as the linear fitting value of the data corresponding to the second stage of the cumulative water absorption curve [44]. The relationship between the rate of capillary water absorption and the curing system of WPUMRC is shown in Figure 7 and Figure 8. Overall, for DC, both the *S*_1_ and *S*_2_ of the WPUMRC decreased with the increase in the standard curing time, reaching the minimum value under the curing system of 28 d standard curing—0 d indoor natural drying curing. For HC, both the *S*_1_ and *S*_2_ of the WPUMRC first decreased and then increased with the increase in the standard curing time, reaching the minimum value under the curing system of 14 d standard curing—14 d indoor natural drying curing. This is consistent with the relationship between cumulative water absorption and the curing system. 

The ratio of *S*_1_–*S*_2_ is shown in Table 7. It can be seen that *S*_1_ is significantly larger than *S*_2_. Compared with *S*_2_, *S*_1_ better shows the influences of different factors on the capillary water absorption performance of cement-based materials [45]. Therefore, *S*_1_ is more important for evaluating the capillary water absorption performance of WPUMRC. Then, only *S*_1_ will be considered in the subsequent analysis of the effects of the polymer-cement ratio and the waterborne polyurethane addition method on the capillary water absorption of WPUMRC.

#### 3.2.2. Effect of Polymer-Cement Ratio on the Rate of Capillary Water Absorption

**Figure 9:**
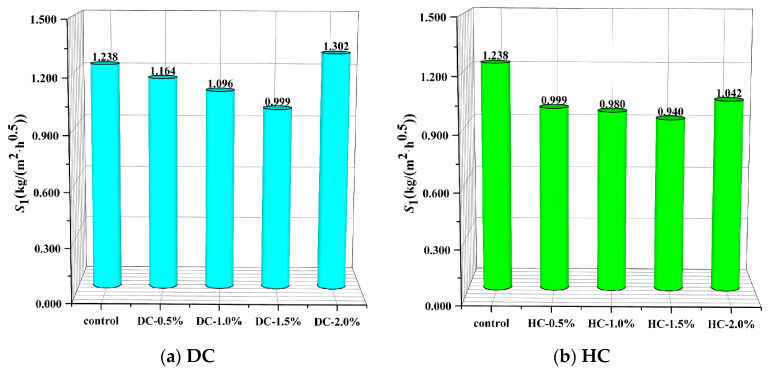
The relationship between the initial rate of absorption and polymer-cement ratio: (**a**) DC, (**b**) HC.

Under the optimal curing system, the relationship between the *S*_1_ and polymer-cement ratio of WPUMRC is shown in Figure 9. For DC, compared with the control, the maximum reduction in the *S*_1_ of WPUMRC was 19.31%; for HC, the maximum reduction in the *S*_1_ of WPUMRC was 24.07%. In general, whether DC or HC is adopted, the content of waterborne polyurethane should be less than 2.0%, which is more conducive to improving the capillary water absorption performance of WPUMRC and possesses lower economic costs.

#### 3.2.3. Effect of Addition Method on the Rate of Capillary Water Absorption

**Figure 10:**
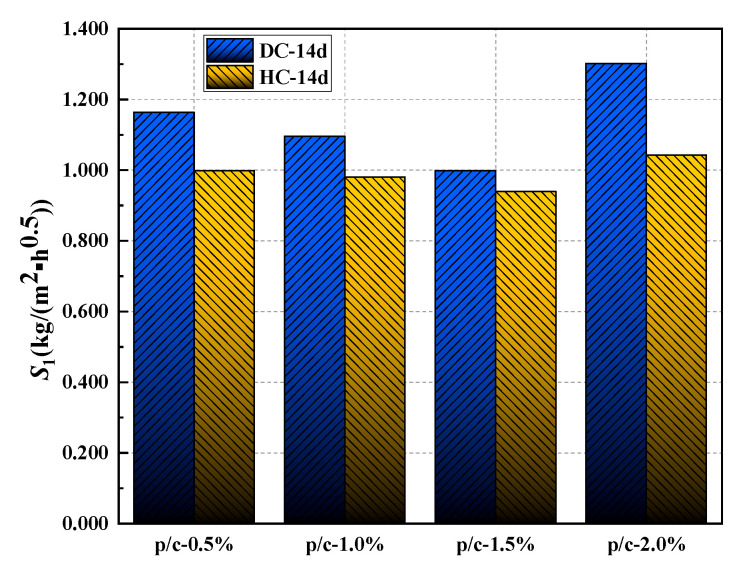
The effect of waterborne polyurethane addition method on the initial rate of absorption.

Under the optimal curing system, the effect of the waterborne polyurethane addition method on the initial rate of capillary water absorption of WPUMRC is shown in Figure 10. It can be seen that the initial rate of capillary water absorption of WPUMRC was lower for HC. Therefore, the waterborne polyurethane addition method of HC is more conducive to improving the capillary water absorption performance of WPUMRC.

## 4. Prediction of Capillary Water Absorption

The cumulative water absorption is the sum of the water absorption mass per unit area of the tested concrete material during the capillary water absorption test, described in the cumulative water absorption curve. The cumulative water absorption curve can reflect the capillary water absorption trend of the concrete material. If gravity can be neglected, the variation in the cumulative water absorption over time can be described using the following equation [46]:

(1) $$\Delta W=S\sqrt{t}$$

where Δ*W* is the cumulative water absorption per unit area in kg/m^2^, *t* is the time in h^0.5^, and *S* is the rate of capillary water absorption in kg/(m^2^·h^0.5^).

The above model assumes that the capillary pores in concrete are horizontal and that the water absorption state is one-dimensional. The model also ignores the effect of gravity on the cumulative water absorption and therefore has the disadvantage of not considering gravity factors. If the effect of gravity is considered, the exponential relationship derived from the experience of Hoffman and Niesel [47,48] is used to describe the relationship between the capillary water absorption per unit area of WPUMRC specimens and the square root of time [42]:

(2) $$\Delta W=a\left[1-e^{-b\sqrt{t}}\right]$$

where *a* and *b* are parameters, which can be obtained by fitting Equation (2) with the experimental data. Through the fitting, it can be seen that the experimental data and formula fit well.

This model does not take into account the change in the water transmission mode in the calculation and ignores the diffusion amount. This is due to the fact that capillary adsorption dominates in water transport when the material is initially dry or partially saturated. As water continues to soak into the interior of the material, it slowly approaches saturation. As water encounters smaller gel pores and other impermeable pores, the mode of water transmission changes and begins to be dominated by diffusion [49].

Through fitting, it can be seen that the experimental data and formula fit well. Introducing the polymer-cement ratio factor, it is found that the relationship between the parameters *a* and *b* and the polymer-cement ratio can be described using the following equations:

(3) $$a=c_{1}p^{3}+d_{1}p^{2}+e_{1}p+f_{1}$$

(4) $$b=c_{2}p^{2}+d_{2}p+e_{2}$$

where *p* is the polymer-cement ratio, and *c*_1_/*c*_2_, *d*_1_/*d*_2_, *e*_1_/*e*_2_, and *f*_1_ are parameters, which are obtained by fitting Equations (3) and (4) with the experimental data, as shown in Table 8 and Table 9.

Ultimately, considering both the polymer-cement ratio and time factors, the cumulative water absorption of WPUMRC is described using Equation (5):

(5) $$\Delta W=\left(c_{1}p^{3}+d_{1}p^{2}+e_{1}p+f_{1}\right)\left[1-e^{-\left(c_{2}p^{2}+d_{2}p+e_{2}\right)\sqrt{t}}\right]$$

In order to compare the effects of parameters *a* and *b* on the model, the following calculations are performed. First, to determine the degree of the effect of parameter *a* on the model, *b* is treated as a fixed constant, and *a* is amplified by n times to calculate Δ*W*′. The difference with the previous Δ*W* is calculated to obtain Δ_1_. Similarly, to determine the degree of the effect of parameter *b* on the model, *a* is treated as a fixed constant, and *b* is amplified by n times to calculate Δ*W*′. The difference with the previous ΔW is calculated to obtain Δ*W*″, and the difference with the previous Δ*W* is calculated to obtain Δ_2_.

Through the calculation of all groups, the absolute value of Δ_1_ is always greater than Δ_2_. This shows that the effect of parameter *a* on the model is greater than the effect of parameter *b* on the model. Considering the large amount of calculation data, Δ_1_ and Δ_2_ are listed for DC, with *n* = 0.3, 0.5, 0.8, 1.5, 2, 2.5, and 3, a polymer-cement ratio of 0.5%, and the curing system of 14 d standard curing—14 d indoor natural drying curing. As shown in Table 10, the absolute value of Δ_1_ is between 0.047 and 13.183%, and the absolute value of Δ_2_ is between 0.046 and 2.908%.

After analyzing the effects of parameters *a* and *b* on the model, the effects of *c*, *d*, *e*, and *f* on parameters *a* and *b* are analyzed using the same method via Equations (3) and (4). The results show that the effects of *c*, *d*, *e*, and *f* on parameter *a* are *f*, *e*, *d*, and *c* in descending order, and the effects of *c*, *d*, and *e* on parameter *b* are *e*, *d*, and *c* in descending order. Considering the large amount of data, the details are not listed.

## 5. The Correlation between the Capillary Water Absorption Performance and the Pore Structure

### 5.1. Pore Structure Parameters Analysis

Mercury intrusion porosimetry (MIP) is also known as the mercury porosity method. It is based on the principle that, in general, mercury does not wet solids and external pressure is required to make the mercury enter a solid’s pores. The mercury penetrates the solid via pressure, resulting in the energy required for the increase in the pore volume equal to the work performed by the external force. Moreover, the amount of mercury pressed into the material is related to the size and distribution of the pores, while the pressure is related to the size of the pores. Qualitatively speaking, the smaller the pore, the greater the mercury pressure required, and vice versa [50]. MIP has been widely used to measure the pore structure characteristics of concrete materials. The results of the MIP test are influenced by parameters such as the properties of the cement paste itself and contact angle [51]. Therefore, when the pore structure characteristics of different samples are compared using MIP data, all influencing factors (such as the sample preparation method, sample drying method, contact angle, surface tension, etc.) must be consistent [51]. In this experiment, all samples were prepared under the same conditions. Some meaningful parameters obtained from the MIP test are very important for characterizing the pore structure of concrete materials, such as the total porosity, effective porosity, ink-bottle porosity, pore connectivity, pore tortuosity, etc. 

**Figure 11:**
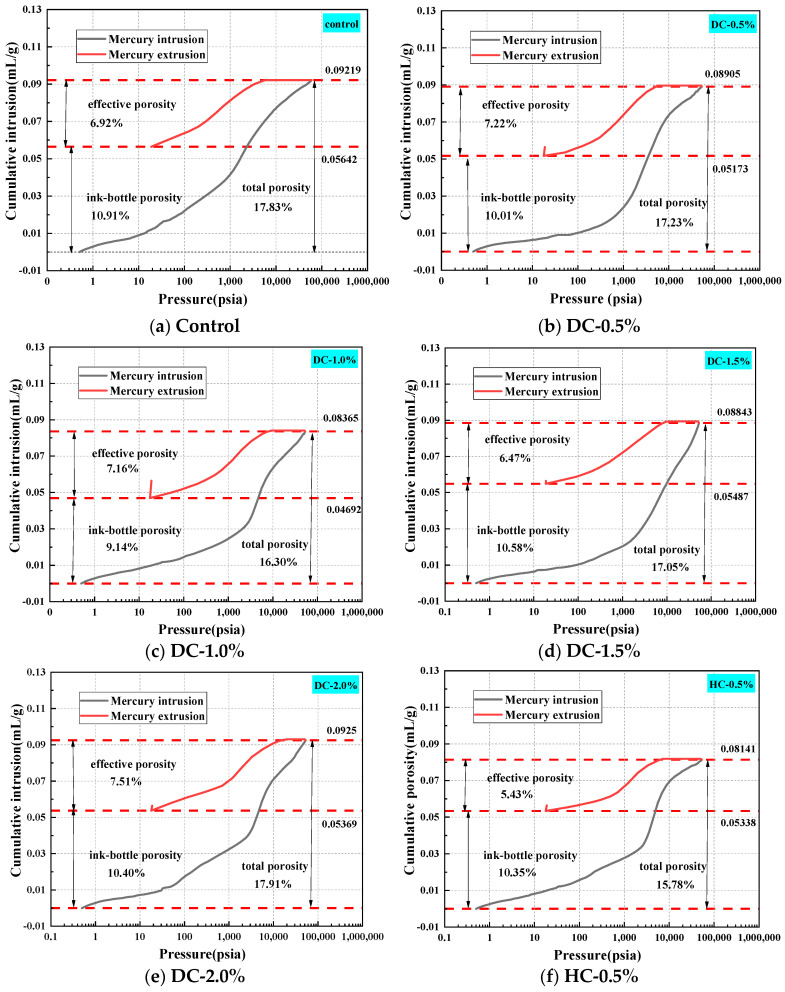

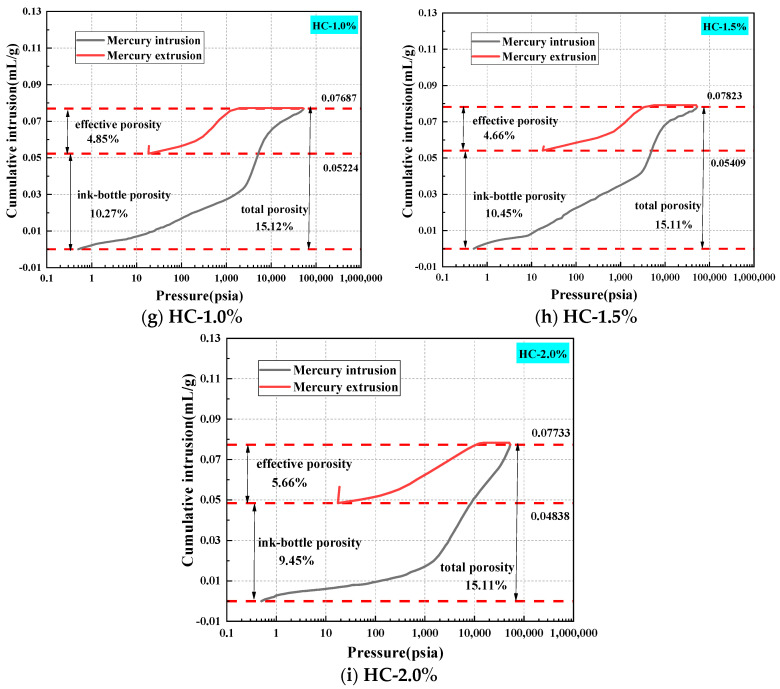
The determination of effective porosity and ink-bottle porosity of different samples. (**a**) control, (**b**) DC-0.5%, (**c**) DC-1.0%, (**d**) DC-1.5%, (**e**) DC-2.0%, (**f**) HC-0.5%, (**g**) HC-1.0%, (**h**) HC-1.5%, (**i**) HC-2.0%.

The total porosity is directly obtained from the MIP report. However, when conducting MIP testing, some assumptions need to be made, one of which is that each pore is connected directly or through larger pores to the specimen surface [27], a criterion that is unlikely to be met by ordinary porous materials. The ink-bottle effect is when a larger pore is preceded by a smaller neck in the intrusion path of the mercury. In other words, intrusion into the wide inner body will not occur until sufficient pressure is applied to force the mercury into the narrow openings [52]. Therefore, some mercury will remain in the sample during the mercury intrusion and extrusion cycles [53]. The total porosity consists of the ink-bottle porosity and effective porosity. The ink-bottle porosity is the ratio of the volume of mercury retained in the pores to the total volume of intrusion mercury, and the effective porosity is the ratio of the volume of unlocked mercury to the total volume of intrusion mercury [54]. The effective porosity plays a more significant role in the transmission of the erosion medium in porous materials [51]. The calculation of the effective porosity and ink-bottle porosity is shown in Figure 11.

The transmission of the erosion medium cannot be carried out via closed pores but instead via connected pores inside concrete materials. Connected pores are important media for fluids and gases to enter concrete materials. Therefore, the smaller the pore connectivity, the weaker the water transport capacity. The pore connectivity [54,55] *η* can be described using Equation (6):

(6) $$\eta =\frac{\Phi_{e}}{\Phi_{t}}\times 100$$

The pole torsion [54,56] *τ* describes the degree of curvature of the pore structure. The pore tortuosity is a parameter defined by the diffusion transfer performance, which can be described using Equations (7) and (8):

(7) $$\tau =4.6242\ln\left(\frac{4.996}{1-\propto_{en}}-1\right)-5.8032$$

(8) $$\propto_{en}=\frac{\Phi_{ink}}{\Phi_{t}}$$

**Figure 12:**
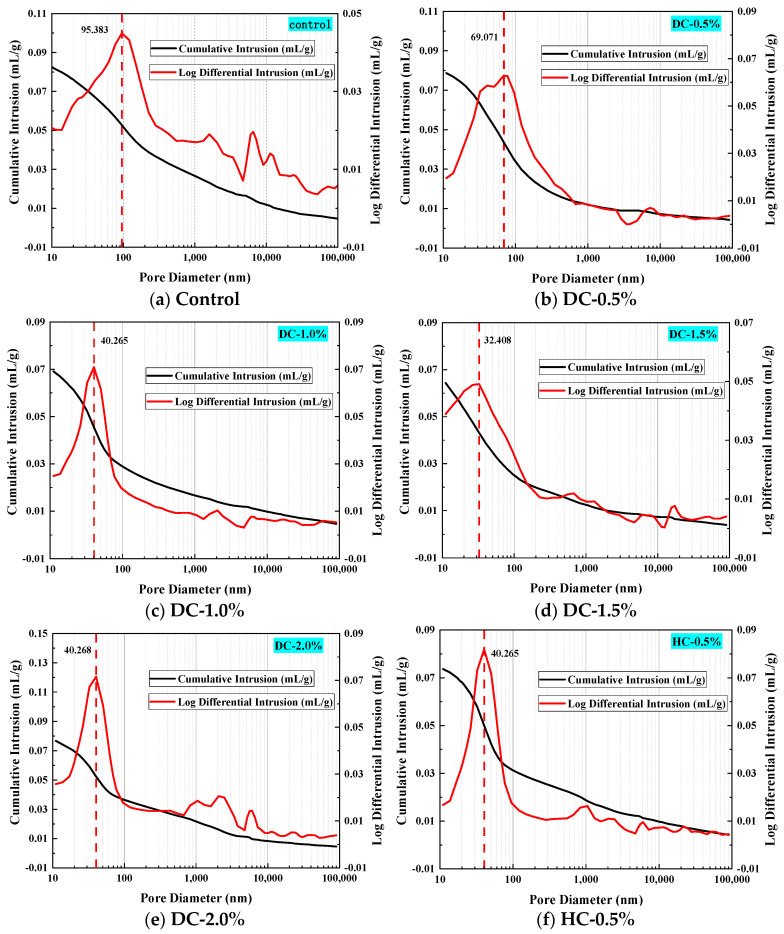

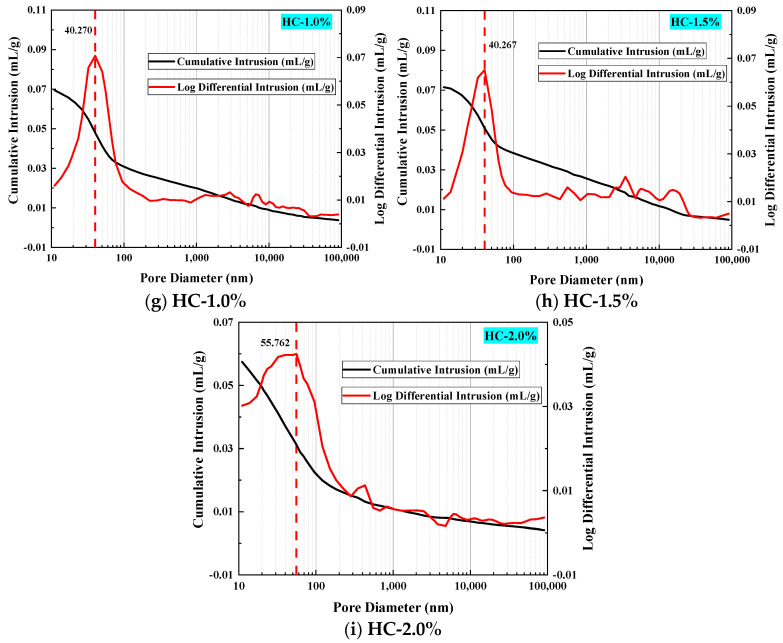
The determination of critical pore diameters of different samples: (**a**–**i**).

The critical pore diameter is one of the most representative and commonly used parameters to establish the relationship between the microstructure and macro performance of cement-based materials. It is also an important parameter to describe transport properties [57]. The critical pore diameter is the largest continuous pore and has a good correlation with permeability [58,59]. Consequently, the impermeability of concrete is improved when the critical pore diameter is reduced. The critical pore diameter can be obtained from Figure 12, which corresponds to the horizontal coordinate value of the peak of the differential curve or that of the steepest slope of the cumulative curve [60].

The average pore diameter is an indicator of the fineness of a porous material [61], which can be calculated using Equation (9):

(9) $$d_{ave}=\frac{4V}{S}$$

where *d*_ave_, *V*, and *S* represent the average pore diameter, total pore volume, and total pore surface area, respectively.

The pore diameter distribution is also an important factor in determining the capillary water absorption performance of WPUMRC. The pores in concrete materials are divided into air voids (>10,000 nm), large capillaries (50~10,000 nm), medium capillaries (10~50 nm), and gel pores (<10 nm) [62]. Among these, gel pores have little influence on the water transmission inside WPUMRC, and capillaries are the main factors for the water transmission inside WPUMRC. 

**Figure 13:**
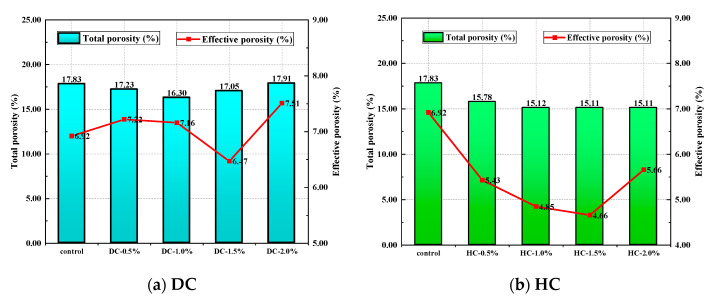
The total porosity and the effective porosity of different samples.

**Figure 14:**
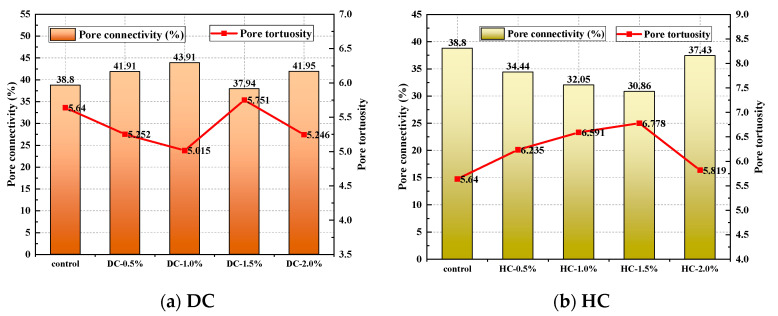
The pore connectivity and the pore tortuosity of different samples.

**Figure 15:**
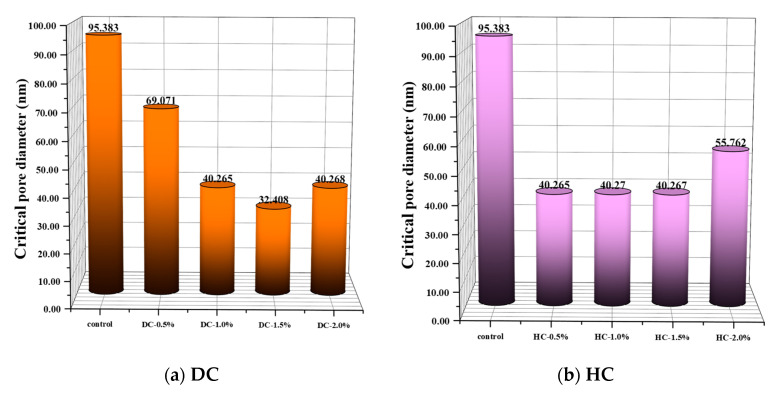
The critical pore diameters of different samples.

**Figure 16:**
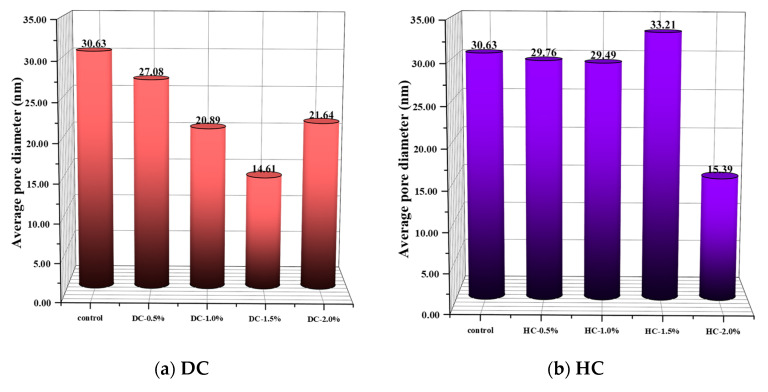
The average pore diameters of different samples.

**Figure 17:**
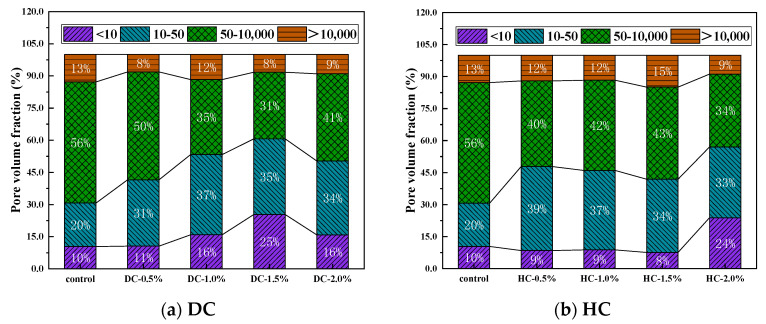
The pore volume fractions of different samples.

The porosity, the pore connectivity, the pore tortuosity, the critical pore diameter, the average pore diameter, and the pore volume fraction of WPUMRC are shown in Figure 13, Figure 14, Figure 15, Figure 16 and Figure 17. For DC, compared with ordinary recycled concrete, the total porosity of the WPUMRC with the dosage of waterborne polyurethane of less than 2.0% was reduced, and the total porosity of the WPUMRC with the dosage of waterborne polyurethane of 2.0% was increased. This is consistent with the conclusion that the initial rate of capillary water absorption of the WPUMRC was similar to that of ordinary recycled concrete. With the increase in the dosage of waterborne polyurethane, the effective porosity, the pore connectivity, and the pore tortuosity of the WPUMRC did not show a clear law. However, for HC, compared with ordinary recycled concrete, the total porosity and the effective porosity of the WPUMRC were significantly reduced, the pore connectivity of the WPUMRC was reduced, and the pore tortuosity of the WPUMRC was increased. Regardless of whether DC or HC was adopted, the critical pore diameter and the average pore diameter of WPUMRC were basically lower than those of the control. In addition, the addition of waterborne polyurethane resulted in an increase in the number of small pores (gel pores and medium capillaries) and a decrease in the number of large pores (large capillaries and air voids). It can be seen that the waterborne polyurethane particles continuously gathered together to become agglomerates and solidify into films under drying curing, the polymer films interweaved with the hydration products of the cement to form a spatial network structure [42], and the waterborne polyurethane agglomerates and films could fill the pores and microcracks inside the WPUMRC, reducing the porosity, increasing the tortuosity of water transfer paths, refining the pore structure, reducing the pore connectivity, and thereby improving the capillary water absorption performance of the WPUMRC. However, it is not possible to directly evaluate the capillary water absorption performance of WPUMRC using a single microscopic indicator. Therefore, it is necessary to comprehensively consider various indicators and establish a relationship between the initial capillary water absorption rate and pore structure parameters.

### 5.2. Grey Relational Grade between the Initial Rate of Capillary Water Absorption and Pore Structure Parameters

The grey correlation analysis (GRA) method is a statistical analysis of multiple factors. GRA uses grey correlation to describe the strength, magnitude, and order of the relationship between factors. The calculation steps mainly include five steps: determining the reference sequence and comparison sequence, data dimensionless processing, calculating the management coefficient between the reference sequence and comparison sequence, and calculating and ranking the correlation degree. The higher the correlation, the higher the level of the evaluation object. In application, the GRA method [63] is always used to measure the correlations between different factors. The reference sequence (i.e., the target value) and the comparison sequence (i.e., the impact factor) are needed to calculate the grey relational grade, the value of which is between zero and one. The greater the grey relational grade between a certain impact factor and the target value, the greater the influencing degree of that impact factor on the target value. Generally, when the grey relational coefficient is greater than 0.6, it is considered that the correlation between the comparison sequence and the reference sequence is close. The GRA method has been studied to analyze the influencing factors of slope stability, and the results of this analysis play an important role in slope stability analysis as well as slope design and reinforcement [64]. The basic principle and calculation steps of the grey relational analysis (GRA) method are as follows:

(i) Determine the original sequences.

The reference sequence (i.e., the target value):

$$X_{0}=\left[X_{0}\left(1\right),X_{0}\left(2\right),\cdots X_{0}\left(k\right)\right]$$

The comparison sequence (i.e., the impact factor):

$$X_{i}=\left[X_{i}\left(1\right),X_{i}\left(2\right),\cdots X_{i}\left(k\right)\right],i=1,2,\cdots n$$

(ii) Carry out the dimensionless processing of the original sequences.

The advantage of dimensionless methods is that they can reduce the dimensional difference between indicators. Dimensionless methods are mainly divided into linear and nonlinear dimensionless processing. Among these, linear dimensionless methods do not change the distribution characteristics of the data. The commonly used linear dimensionless methods include the scaling method, translation method, normalization method, averaging method, etc. The averaging method was selected in this article, as shown in Equation (10).

(10) $$Y_{i}\left(k\right)=\frac{X_{i}\left(k\right)}{\frac{1}{k}\sum_{1}^{k}X_{i}\left(k\right)}$$

(iii) Calculate the grey relational coefficient and grey relational grade, and then determine the grey relational order.

(11) $$C_{i}\left(k\right)=\left|X_{0}\left(k\right)-X_{i}\left(k\right)\right|$$

(12) $$\varepsilon_{0i}\left(k\right)=\frac{\delta_{min}+\rho \delta_{max}}{C_{i}\left(k\right)+\rho \delta_{max}}$$

(13) $$\delta_{min}=\underset{i}{\min}\;\underset{k}{\min}\;C_{i}\left(k\right)$$

(14) $$\delta_{max}=\underset{i}{\max}\;\underset{k}{\max}\;C_{i}\left(k\right)$$

(15) $$r_{0i}=\frac{1}{k}\overset{k}{\underset{1}{\sum }}\varepsilon_{0i}\left(k\right)$$

where ε_0*i*_ is the grey relational coefficient. *ρ* is the resolution ratio, the value of which is usually 0.5. *r*_0*i*_ is the grey relational grade.

The grey relational grade between different pore structural parameters and the initial rate of capillary water absorption are shown in Table 11, Table 12, Table 13, Table 14 and Table 15. The grey relational grade between all micro indicators and the initial rate of capillary water absorption is greater than 0.6, indicating that the capillary water absorption performance of WPUMRC is closely related to its pore structure. The effect levels of the pore structure parameters on the capillary water absorption performance of WPUMRC from high to low are *Φ*_t_, *η*, *Φ*_e_, *τ*, *d*_cri_, and *d*_ave_, respectively.

### 5.3. Quantitative Relationship between the Initial Rate of Capillary Water Absorption and the Pore Structure Parameters

The grey correlation analysis between the pore structure parameters and initial capillary water absorption showed that the capillary water absorption performance of WPUMRC is correlated with all pore structure indicators. Therefore, a quantitative relationship between the capillary water absorption performance and each pore structure parameter needed to be established. A multivariate linear equation was proposed to describe the functional relationship between the initial capillary water absorption *S*_1_ and the pore structure characterization parameters, as shown in Equation (16).

(16) $$S_{1}=x_{1}+x_{2}\cdot \Phi_{t}+x_{3}\cdot \Phi_{e}+x_{4}\cdot \eta +x_{5}\cdot \tau +x_{6}\cdot d_{\mathrm{ave}}+x_{7}\cdot d_{\mathrm{cri}}$$

where *x*_1_, *x*_2_, *x*_3_, *x*_4_, *x*_5_, *x*_6_, and *x*_7_ are the coefficients.

The effective porosity, total porosity, and pore connectivity are not independent parameters, and their relationship with each other can be described using Equation (17) [54,55]. The pore connectivity and pore tortuosity are not independent parameters, and their relationship can be described using Equation (18) [54,55]. The effective porosity and average pore diameter are not independent parameters, and their relationship with each other can be described using Equation (19) [61]. Therefore, Equation (16) can be further simplified to Equation (20).

(17) $$\Phi_{e}=\Phi_{t}\cdot \eta$$

(18) $$\tau =4.6242\ln\left(\frac{4.996}{\eta }-1\right)-5.8032$$

(19) $$d_{\mathrm{ave}}=\frac{4\cdot v_{t}}{S}=\frac{4\cdot \Phi_{t}}{S\cdot \rho_{\mathrm{bulk}}}$$

(20) $$S_{1}=y_{1}+y_{2}\cdot \Phi_{t}+y_{3}\cdot \eta +y_{4}\cdot d_{\mathrm{cri}}$$

where *y*_1_, *y*_2_, *y*_3_, and *y*_4_ are the coefficients.

Equation (20) simultaneously contains the porosity parameter (total porosity), the pore size characteristics (critical pore size), and the pore network characteristics (pore connectivity), which can better summarize the relationship between the capillary water absorption performance and the pore structure characteristics of WPUMRC.

Combined with the test data, the coefficients in Equation (20) can be calculated, and then the quantitative relationship between the capillary water absorption performance and the internal pore structure of the WPUMRC is expressed in Equation (21):

(21) $$S_{1}=-0.2494+0.0574\cdot \Phi_{t}+0.0085\cdot \eta +0.0015\cdot d_{\mathrm{cri}}$$

The errors between the theoretical and test values were calculated, as shown in Table 16. It can be seen that the error between the test and theoretical values is relatively small, with an error range of 0.2~9.1%. This indicates that the proposed theoretical model can effectively evaluate the relationship between the capillary water absorption performance and the pore structure of WPUMRC.

## 6. Conclusions

The capillary water absorption performance of WPUMRC under different curing systems, polymer-cement ratios, and waterborne polyurethane addition methods was studied in this article. Based on the MIP testing results, the relationship between the capillary water absorption and pore structure of WPUMRC under the optimal curing system was established using the grey relational method. The following conclusions are drawn:

(1) As the standard curing time increased, the cumulative water absorption of WPUMRC gradually decreased for DC. The cumulative water absorption of WPUMRC first decreased and then increased for HC, reaching the minimum value under the system of 14 d standard curing—14 d indoor natural drying curing. The changes in the *S*_1_ and *S*_2_ of WPUMRC with the curing systems were basically consistent with the changes in the cumulative water absorption with the curing systems. Taking into account the compressive strength and capillary water absorption performance of WPUMRC, whether DC or HC is adopted, the optimal curing system is the system of 14 d standard curing—14 d indoor natural drying curing.

(2) Under the optimal curing systems, compared with the control, the cumulative water absorption and the initial rate of capillary water absorption of WPUMRC both decreased, except for the WPUMRC with the polymer-cement ratio of 2.0% for DC. Therefore, considering the economic cost and capillary water absorption performance of WPUMRC, the dosage of waterborne polyurethane should be less than 2.0%.

(3) According to the test results, the prediction model of the capillary water absorption of recycled concrete was established with the polymer-cement ratio and the square root of the capillary water absorption time as variables. The experimental results are in good agreement with the proposed model, which can provide a reference for the study of the capillary water absorption performance of polymer recycled concrete.

(4) The MIP test results indicate that the micro modification mechanism of waterborne polyurethane in recycled concrete is as follows: waterborne polyurethane can fill the pores and microcracks inside WPUMRC or interweave with the hydration products of cement to form a spatial network structure, reducing the pore connectivity, increasing the tortuosity of water transfer paths, refining the pore structure, reducing the porosity, and thereby improving the capillary water absorption performance of the WPUMRC.

(5) The grey relational grades of the different pore structure parameters on the *S*_1_ of WPUMRC from high to low were *Φ*_t_, *η*, *Φ*_e_, *τ*, *d*_cri_, and *d*_ave_, respectively. It is not possible to directly evaluate the capillary water absorption performance of WPUMRC using a single microscopic indicator. Therefore, the relationship between the *S*_1_ and various pore structure parameters based on the grey relational method was established.

## Figures and Tables

**Table 1:** Basic parameters of P·O 42.5 cement.

		Setting Time (min)		Compressive Strength (MPa)		
Cement Grade	Specific Gravity (g/cm^3^)	Initial Setting	Final Setting	3 d	28 d	Specific Surface (m^2^/kg)
P·O 42.5	3.14	185	295	28.8	48.2	316

**Table 2:** Physical properties of recycled coarse aggregate.

Particle Size (mm)	Water Absorption (%)	Apparent Density (kg/m^3^)	Bulk Density (kg/m^3^)	Void Fraction (%)	Crushing Index Value (%)
5~20	5.32	2586	1333	49	18.8

**Table 3:** Basic parameters of waterborne polyurethane emulsion.

Color	Solid Content	pH	Softening Point	Viscosity
White	50%	7.5–10	154.44 °C	200–1000 mPa·s

**Table 4:** Basic parameters of organosilicon antifoaming agent.

Color	Solid Content	Viscosity (25 °C)	Diluent
White	30%	2500 mPa·s	Water

**Table 5:** Mix design of concrete (kg/m^3^).

Specimen	Cement	Fine Aggregate	10–20 mm Coarse Aggregate	5–10 mm Coarse Aggregate	Polycarboxylate Superplasticizer	Waterborne Polyurethane	Antifoaming Agent	Effective Water	Additional Water
Control	461.76	629.41	750.00	320.59	4.62	0.00	0.00	194.12	33.19
p/c-0.5%	461.76	629.41	750.00	320.59	4.62	2.31	0.09	192.89	33.19
p/c-1.0%	461.76	629.41	750.00	320.59	4.62	4.62	0.18	191.68	33.19
p/c-1.5%	461.76	629.41	750.00	320.59	4.62	6.93	0.28	190.46	33.19
p/c-2.0%	461.76	629.41	750.00	320.59	4.62	9.24	0.37	188.78	33.19

**Table 6:** Test time and tolerance time.

**Time**	60 s	5 min	10 min	20 min	30 min	1 h	2 h	3 h	4 h	5 h	6 h	8 h	1 d	2 d	3 d	4 d	5 d
**Tolerance**	2 s	10 s	2 min	2 min	2 min	2 min	5 min	5 min	5 min	5 min	5 min	10 min	2 h	2 h	2 h	2 h	2 h

**Table 7:** The ratio of initial water absorption to second water absorption (*S*_1_/*S*_2_).

	Addition Method	Polymer-Cement Ratio	Curing System				
			0 d	3 d	7 d	14 d	28 d
	Control	0	3.229	3.587	3.064	3.057	3.101
*S*_1_/*S*_2_	DC	0.5%	2.406	3.505	2.846	3.096	2.897
		1.0%	3.005	2.927	2.966	2.747	2.811
		1.5%	2.122	2.644	2.362	2.112	2.394
		2.0%	1.691	2.420	2.048	3.168	2.610
	HC	0.5%	2.994	3.145	2.445	3.112	3.318
		1.0%	2.808	3.095	2.417	2.361	2.376
		1.5%	1.753	2.863	2.750	1.983	2.118
		2.0%	2.460	2.154	2.179	2.122	2.050

**Table 8:** WPUMRC *a* and *b* values.

Addition Method	Polymer-Cement Ratio	Fitting Parameter	Curing System				
			0 d	3 d	7 d	14 d	28 d
Control	0	*a*	15.676	11.541	9.36	7.306	6.742
		*b*	0.214	0.27	0.248	0.285	0.303
DC	0.5%	*a*	12.869	9.709	7.456	6.974	6.943
		*b*	0.177	0.255	0.237	0.265	0.281
	1.0%	*a*	13.177	10.126	8.546	7.059	6.750
		*b*	0.184	0.200	0.214	0.250	0.246
	1.5%	*a*	16.406	9.739	8.842	7.911	7.204
		*b*	0.115	0.226	0.199	0.178	0.204
	2.0%	*a*	17.490	11.755	10.911	7.532	7.407
		*b*	0.105	0.175	0.165	0.278	0.223
HC	0.5%	*a*	12.657	10.820	8.430	5.758	7.134
		*b*	0.222	0.273	0.230	0.294	0.302
	1.0%	*a*	13.611	11.656	8.931	6.565	6.806
		*b*	0.201	0.258	0.218	0.229	0.255
	1.5%	*a*	18.723	10.309	10.108	7.516	7.486
		*b*	0.117	0.268	0.222	0.180	0.239
	2.0%	*a*	17.451	12.259	12.059	7.417	8.344
		*b*	0.154	0.155	0.148	0.213	0.192

**Table 9:** WPUMRC *c*_1_, *c*_2_, *d*_1_, *d*_2_, *e*_1_, *e*_2_, and *f*_1_ values.

Addition Method	Curing System	Parameter	Fitting Parameter			
			*c*_1_, *c*_2_	*d*_1_, *d*_2_	*e*_1_, *e*_2_	*f*_1_
DC	0 d	*a*	−3.507	13.578	−12.222	15.745
		*b*	−0.006	−0.044	0.212	-
	3 d	*a*	0.103	1.661	−3.626	11.452
		*b*	0.003	−0.049	0.270	-
	7 d	*a*	−0.814	4.484	−4.938	9.269
		*b*	−0.011	−0.020	0.248	-
	14 d	*a*	−1.098	3.487	−2.468	7.339
		*b*	-	-	-	-
	28 d	*a*	0.095	−0.100	0.151	6.770
		*b*	0.022	−0.091	0.310	-
HC	0 d	*a*	−6.905	22.900	−17.294	15.829
		*b*	-	-	-	-
	3 d	*a*	-	-	-	-
		*b*	−0.059	0.071	0.262	-
	7 d	*a*	−0.438	3.152	−3.204	9.347
		*b*	−0.027	0.013	0.241	-
	14 d	*a*	−2.270	7.678	−6.222	7.291
		*b*	0.018	−0.088	0.301	-
	28 d	*a*	0.599	−1.242	0.890	6.779
		*b*	−0.017	−0.023	0.307	-

**Table 10:** The degrees of effects of *a* and *b* on Δ*W*.

n	*T*^0.5^ (h^0.5^)	Δ*W* (kg/m^2^)	Δ*W*′ (kg/m^2^)	Δ*W*″ (kg/m^2^)	Δ_1_ (kg/m^2^)	Δ_2_ (kg/m^2^)
0.3	0.12911	0.23457	0.046915	0.071217	−0.18766	−0.16336
	0.28867	0.51360	0.10272	0.158226	−0.41088	−0.35537
	0.40825	0.71511	0.143022	0.222714	−0.57209	−0.4924
	0.57735	0.98939	0.197878	0.312867	−0.79151	−0.67652
	0.70711	1.19168	0.238336	0.381229	−0.95334	−0.81045
	1	1.62351	0.324701	0.532967	−1.2988	−1.09054
	1.41421	2.17972	0.435944	0.741614	−1.74378	−1.43811
	1.73205	2.56699	0.513399	0.897123	−2.05359	−1.66987
	2	2.86907	0.573814	1.025203	−2.29526	−1.84387
	2.23607	3.11800	0.6236	1.135807	−2.4944	−1.98219
	2.44949	3.33003	0.666006	1.234027	−2.66402	−2.096
	2.82843	3.67818	0.735636	1.404369	−2.94254	−2.27381
	4.89898	5.07000	1.013999	2.249697	−4.056	−2.8203
	6.9282	5.86194	1.172388	2.953537	−4.68955	−2.9084
	8.48528	6.23792	1.247583	3.421651	−4.99033	−2.81626
	9.79796	6.45418	1.290836	3.773679	−5.16334	−2.6805
	10.95445	6.59139	1.318278	4.054798	−5.27311	−2.53659
0.5	0.12911	0.23457	0.117287	0.11829	−0.11729	−0.11628
	0.28867	0.51360	0.256799	0.26171	−0.2568	−0.25189
	0.40825	0.71511	0.357555	0.367224	−0.35756	−0.34789
	0.57735	0.98939	0.494695	0.513608	−0.49469	−0.47578
	0.70711	1.19168	0.595841	0.623733	−0.59584	−0.56795
	1	1.62351	0.811753	0.865453	−0.81175	−0.75805
	1.41421	2.17972	1.089861	1.191673	−1.08986	−0.98805
	1.73205	2.56699	1.283497	1.430133	−1.2835	−1.13686
	2	2.86907	1.434534	1.623506	−1.43453	−1.24556
	2.23607	3.11800	1.559	1.788275	−1.559	−1.32972
	2.44949	3.33003	1.665014	1.932864	−1.66501	−1.39717
	2.82843	3.67818	1.83909	2.179728	−1.83909	−1.49845
	4.89898	5.07000	2.534998	3.330029	−2.535	−1.73997
	6.9282	5.86194	2.930969	4.189127	−2.93097	−1.67281
	8.48528	6.23792	3.118958	4.708287	−3.11896	−1.52963
	9.79796	6.45418	3.227089	5.069996	−3.22709	−1.38418
	10.95445	6.59139	3.295695	5.340501	−3.2957	−1.25089
0.8	0.12911	0.23457	0.187659	0.188299	−0.04691	−0.04627
	0.28867	0.51360	0.410879	0.413998	−0.10272	−0.0996
	0.40825	0.71511	0.572089	0.57821	−0.14302	−0.1369
	0.57735	0.98939	0.791512	0.803433	−0.19788	−0.18596
	0.70711	1.19168	0.953345	0.970866	−0.23834	−0.22081
	1	1.62351	1.298805	1.33228	−0.3247	−0.29123
	1.41421	2.17972	1.743777	1.806565	−0.43594	−0.37316
	1.73205	2.56699	2.053595	2.143286	−0.5134	−0.42371
	2	2.86907	2.295255	2.410048	−0.57381	−0.45902
	2.23607	3.11800	2.4944	2.632838	−0.6236	−0.48516
	2.44949	3.33003	2.664023	2.824877	−0.66601	−0.50515
	2.82843	3.67818	2.942544	3.14516	−0.73564	−0.53302
	4.89898	5.07000	4.055996	4.505514	−1.014	−0.56448
	6.9282	5.86194	4.68955	5.36854	−1.17239	−0.4934
	8.48528	6.23792	4.990332	5.819913	−1.24758	−0.418
	9.79796	6.45418	5.163343	6.100265	−1.29084	−0.35391
	10.95445	6.59139	5.273112	6.290244	−1.31828	−0.30115
1.5	0.12911	0.23457	0.351861	0.348885	0.117287	0.114311
	0.28867	0.51360	0.770398	0.756035	0.256799	0.242436
	0.40825	0.71511	1.072666	1.04468	0.357555	0.329569
	0.57735	0.98939	1.484085	1.430133	0.494695	0.440743
	0.70711	1.19168	1.787522	1.708834	0.595841	0.517153
	1	1.62351	2.435259	2.287486	0.811753	0.663981
	1.41421	2.17972	3.269582	2.998937	1.089861	0.819216
	1.73205	2.56699	3.85049	3.470722	1.283497	0.903729
	2	2.86907	4.303603	3.824672	1.434534	0.955604
	2.23607	3.11800	4.677	4.106756	1.559	0.988757
	2.44949	3.33003	4.995043	4.339966	1.665014	1.009937
	2.82843	3.67818	5.51727	4.70829	1.83909	1.03011
	4.89898	5.07000	7.604993	5.979142	2.534998	0.909147
	6.9282	5.86194	8.792907	6.529929	2.930969	0.667991
	8.48528	6.23792	9.356873	6.734861	3.118958	0.496945
	9.79796	6.45418	9.681268	6.832081	3.227089	0.377902
	10.95445	6.59139	9.887086	6.884383	3.295695	0.292992
2	0.12911	0.23457	0.469147	0.461257	0.234574	0.226684
	0.28867	0.51360	1.027198	0.989374	0.513599	0.475775
	0.40825	0.71511	1.430222	1.356895	0.715111	0.641784
	0.57735	0.98939	1.97878	1.838417	0.98939	0.849027
	0.70711	1.19168	2.383362	2.179734	1.191681	0.988053
	1	1.62351	3.247011	2.869069	1.623506	1.245563
	1.41421	2.17972	4.359442	3.678171	2.179721	1.49845
	1.73205	2.56699	5.133987	4.189127	2.566993	1.622133
	2	2.86907	5.738138	4.557817	2.869069	1.688748
	2.23607	3.11800	6.236	4.841976	3.118	1.723976
	2.44949	3.33003	6.660057	5.069996	3.330029	1.739967
	2.82843	3.67818	7.35636	5.416439	3.67818	1.738259
	4.89898	5.07000	10.13999	6.454179	5.069996	1.384183
	6.9282	5.86194	11.72388	6.796673	5.861938	0.934735
	8.48528	6.23792	12.47583	6.896309	6.237915	0.658393
	9.79796	6.45418	12.90836	6.935254	6.454179	0.481075
	10.95445	6.59139	13.18278	6.953009	6.59139	0.361619
2.5	0.12911	0.23457	0.586434	0.571724	0.351861	0.33715
	0.28867	0.51360	1.283997	1.213956	0.770398	0.700357
	0.40825	0.71511	1.787777	1.65267	1.072666	0.937559
	0.57735	0.98939	2.473475	2.216632	1.484085	1.227242
	0.70711	1.19168	2.979203	2.608518	1.787522	1.416837
	1	1.62351	4.058764	3.378479	2.435259	1.754973
	1.41421	2.17972	5.449303	4.241342	3.269582	2.061621
	1.73205	2.56699	6.417483	4.76021	3.85049	2.193217
	2	2.86907	7.172672	5.12029	4.303603	2.251221
	2.23607	3.11800	7.795	5.38867	4.677	2.27067
	2.44949	3.33003	8.325072	5.597696	4.995043	2.267667
	2.82843	3.67818	9.19545	5.903256	5.51727	2.225076
	4.89898	5.07000	12.67499	6.702389	7.604993	1.632394
	6.9282	5.86194	14.65485	6.903189	8.792907	1.041251
	8.48528	6.23792	15.59479	6.94876	9.356873	0.710844
	9.79796	6.45418	16.13545	6.963422	9.681268	0.509243
	10.95445	6.59139	16.47848	6.969083	9.887086	0.377693
3	0.12911	0.23457	0.703721	0.680317	0.469147	0.445743
	0.28867	0.51360	1.540797	1.430111	1.027198	0.916512
	0.40825	0.71511	2.145333	1.93287	1.430222	1.217759
	0.57735	0.98939	2.96817	2.566993	1.97878	1.577603
	0.70711	1.19168	3.575043	2.998953	2.383362	1.807272
	1	1.62351	4.870517	3.824672	3.247011	2.201167
	1.41421	2.17972	6.539163	4.708281	4.359442	2.52856
	1.73205	2.56699	7.70098	5.214184	5.133987	2.647191
	2	2.86907	8.607207	5.551823	5.738138	2.682754
	2.23607	3.11800	9.354	5.795181	6.236	2.677181
	2.44949	3.33003	9.990086	5.979142	6.660057	2.649114
	2.82843	3.67818	11.03454	6.237917	7.35636	2.559737
	4.89898	5.07000	15.20999	6.832081	10.13999	1.762086
	6.9282	5.86194	17.58581	6.945724	11.72388	1.083786
	8.48528	6.23792	18.71375	6.9658	12.47583	0.727884
	9.79796	6.45418	19.36254	6.971112	12.90836	0.516933
	10.95445	6.59139	19.77417	6.972848	13.18278	0.381458

**Table 11:** The reference sequence *X*_0_ and the comparison sequences *X*_i_ of WPUMRC.

		*S*_1_	*Φ*_t_	*Φ*_e_	*η*	*τ*	*d*_ave_	*d*_cri_
		*X*_0_	*X*_1_	*X*_2_	*X*_3_	*X*_4_	*X*_5_	*X*_6_
Samples	1	1.238	17.83	6.92	38.80	5.640	30.63	95.383
	2	1.164	17.23	7.22	41.91	5.252	27.08	69.071
	3	1.096	16.30	7.16	43.91	5.015	20.89	40.265
	4	0.999	17.05	6.47	37.94	5.751	14.61	32.408
	5	1.302	17.91	7.51	41.95	5.246	21.64	40.268
	6	0.999	15.78	5.43	34.44	6.235	29.76	40.265
	7	0.98	15.12	4.85	32.05	6.591	29.49	40.270
	8	0.94	15.11	4.66	30.86	6.778	33.21	40.267
	9	1.042	15.11	5.66	37.43	5.819	15.39	55.762

**Table 12:** Dimensionless processing of the original sequences.

		*S*_1_	*Φ*_t_	*Φ*_e_	*η*	*τ*	*d*_ave_	*d*_cri_
		*Y*_0_	*Y*_1_	*Y*_2_	*Y*_3_	*Y*_4_	*Y*_5_	*Y*_6_
Samples	1	1.142	1.088	1.115	1.029	0.970	1.238	1.891
	2	1.074	1.052	1.163	1.112	0.903	1.094	1.369
	3	1.011	0.995	1.153	1.165	0.863	0.844	0.798
	4	0.922	1.041	1.042	1.006	0.989	0.590	0.643
	5	1.201	1.093	1.210	1.113	0.902	0.875	0.798
	6	0.922	0.963	0.875	0.914	1.072	1.203	0.798
	7	0.904	0.923	0.781	0.850	1.134	1.192	0.798
	8	0.867	0.922	0.751	0.819	1.166	1.342	0.798
	9	0.961	0.922	0.912	0.993	1.001	0.622	1.106

**Table 13:** The difference sequences *C*_i._

		*Φ*_t_	*Φ*_e_	*η*	*τ*	*d*_ave_	*d*_cri_
		*C*_1_	*C*_2_	*C*_3_	*C*_4_	*C*_5_	*C*_6_
Samples	1	0.054	0.027	0.113	0.172	0.096	0.749
	2	0.022	0.089	0.038	0.171	0.020	0.295
	3	0.016	0.142	0.154	0.148	0.167	0.213
	4	0.119	0.120	0.084	0.067	0.332	0.279
	5	0.108	0.009	0.088	0.299	0.326	0.403
	6	0.041	0.047	0.008	0.150	0.281	0.124
	7	0.019	0.123	0.054	0.230	0.288	0.106
	8	0.055	0.116	0.048	0.299	0.475	0.069
	9	0.039	0.049	0.032	0.040	0.339	0.145

**Table 14:** The grey relational coefficients.

		*Φ*_t_	*Φ*_e_	*η*	*τ*	*d*_ave_	*d*_cri_
		*ε*_01_	*ε*_02_	*ε*_03_	*ε*_04_	*ε*_05_	*ε*_06_
Samples	1	0.893	0.952	0.785	0.700	0.813	0.340
	2	0.964	0.826	0.928	0.702	0.969	0.571
	3	0.979	0.740	0.724	0.731	0.707	0.651
	4	0.775	0.773	0.834	0.866	0.542	0.585
	5	0.793	0.999	0.827	0.568	0.546	0.492
	6	0.920	0.906	0.999	0.729	0.584	0.768
	7	0.972	0.769	0.893	0.633	0.578	0.797
	8	0.890	0.779	0.904	0.568	0.450	0.863
	9	0.926	0.902	0.941	0.923	0.536	0.737

**Table 15:** The grey relational grade and the grey relational order.

	*Φ*_t_	*Φ*_e_	*η*	*τ*	*d*_ave_	*d*_cri_
The grey relational grade	0.902	0.850	0.871	0.713	0.636	0.645
The grey relational order	1	3	2	4	6	5

**Table 16:** Error between theoretical and test values.

Specimen	Ordinary Recycled Concrete	DC-0.5%	DC-1.0%	DC-1.5%	DC-2.0%	HC-0.5%	HC-1.0%	HC-1.5%	HC-2.0%
Test value	1.238	1.164	1.096	0.999	1.302	0.999	0.98	0.94	1.042
Theoretical value	1.243	1.196	1.118	1.099	1.193	1.007	0.949	0.939	1.017
Theoretical Value/test value	0.996	0.973	0.981	0.909	1.091	0.992	1.032	1.002	1.025

## Data Availability

Any further specific data analysis can be obtained by making a reasonable request to the corresponding author.
